# Supplementary material for: Exploring the Metabolic Differences between Cisplatin- and UV Light-Induced Apoptotic Bodies in HK-2 Cells by an Untargeted Metabolomics Approach
Source: Int J Mol Sci. 2023 Apr 14;24(8):7237. doi: 10.3390/ijms24087237 (PMC10138416; doi:10.3390/ijms24087237)
Supplement: Supplementary file 1 [file ijms-24-07237-s001.zip › Supporting information.pdf]

EXPLORING THE METABOLIC DIFFERENCES BETWEEN CISPLATIN- AND UV LIGHT-INDUCED APOPTOTIC BODIES IN HK-2 CELLS BY AN UNTARGETED METABOLOMICS APPROACH

Samuel Bernardo-Bermejo[1], Elena Sánchez-López[2], María Castro-Puyana[1,3], Ana B. Fernández-Martínez[4], Francisco Javier Lucio-Cazaña[5] and María Luisa Marina[1,3, *]

[1]Universidad de Alcalá, Departamento de Química Analítica, Química Física e Ingeniería Química, Ctra. Madrid-Barcelona Km.33.600, 28871 Alcalá de Henares (Madrid), Spain.

[2]Center for Proteomics and Metabolomics, Leiden University Medical Center, Albinusdreef 2, 2333ZA Leiden, the Netherlands.

[3]Universidad de Alcalá, Instituto de Investigación Química Andrés M, del Río. Ctra. Madrid-Barcelona Km. 33.600, 28871 Alcalá de Henares (Madrid), Spain.

[4]Departamento de Biología, Universidad Autónoma de Madrid, Madrid, Spain.

[5]Universidad de Alcalá, Departamento de Biología de Sistemas, Ctra. Madrid-Barcelona Km. 33.600, 28871 Alcalá de Henares (Madrid), Spain.

***Correspondence:** Universidad de Alcalá, Departamento de Química Analítica, Química Física e Ingeniería Química, Ctra. Madrid-Barcelona Km.33.600, 28871 Alcalá de Henares (Madrid), Spain.

E-mail: mluisa.marina@uah.es

Fax: +34-91 885 4971

Tel: +34-91 885 4935

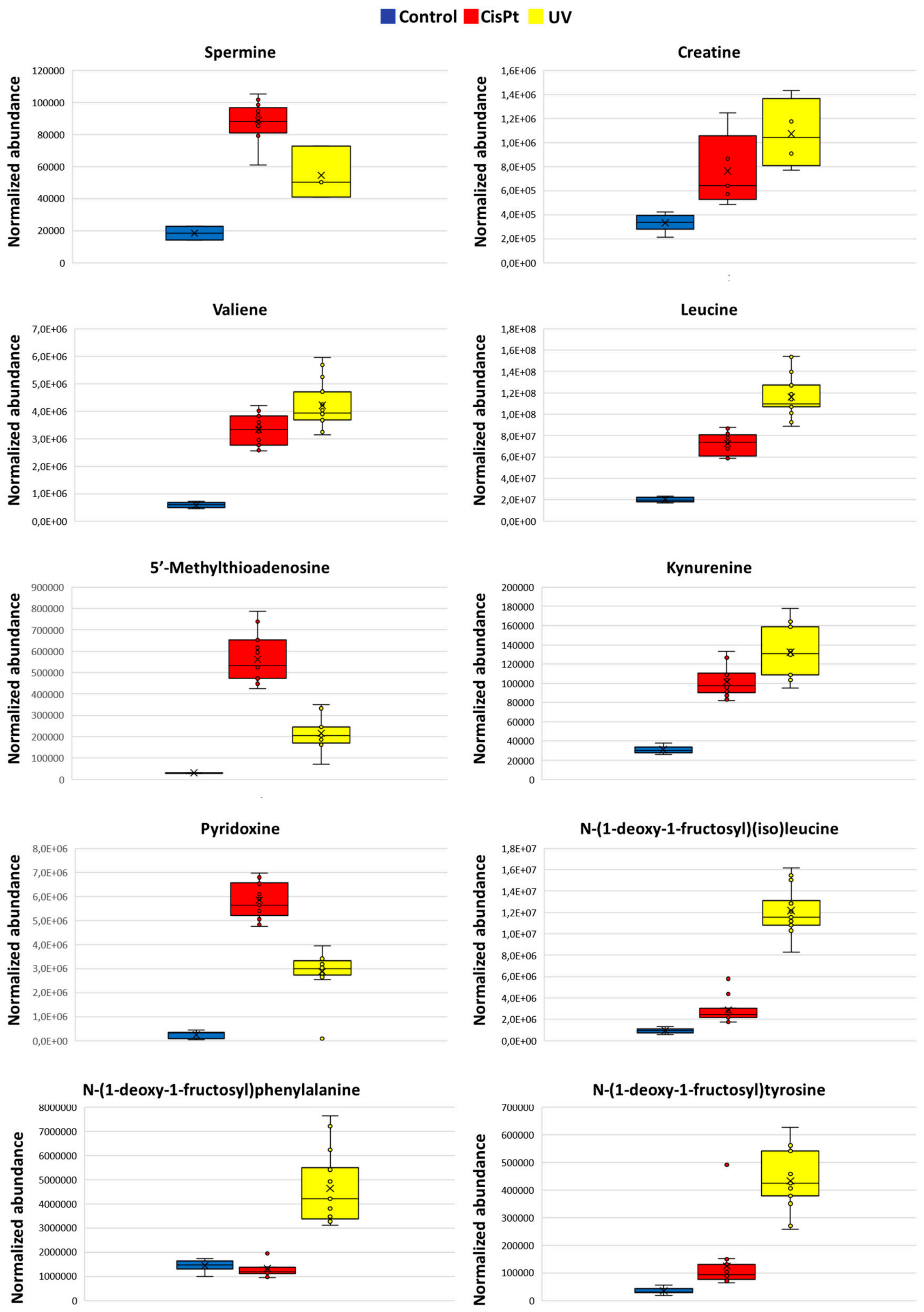

Figure S1. Box-plots of the metabolites identified in the ABs fluid.

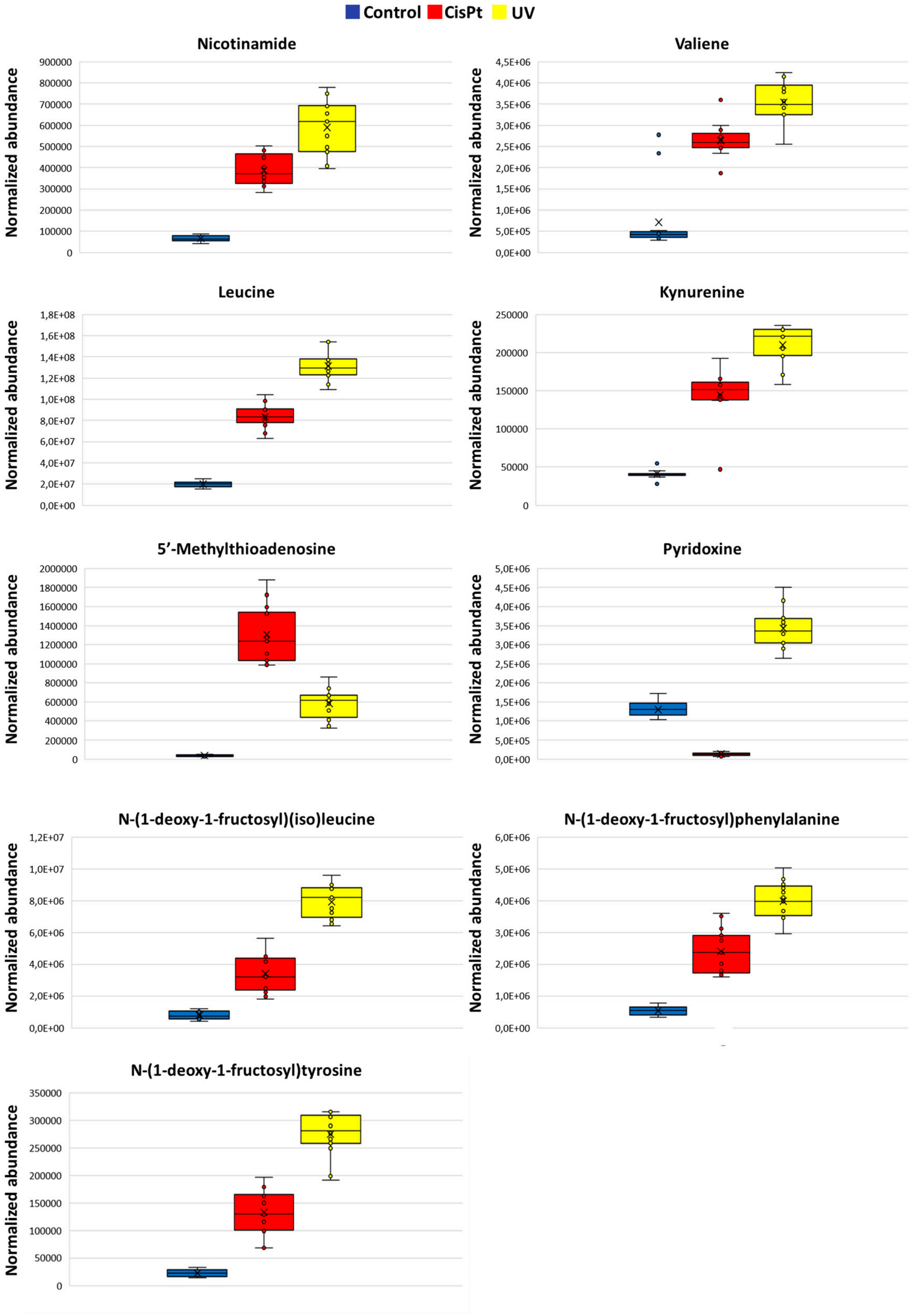

Figure S2. Box-plots of the metabolites identified in the extracellular fluid.

Table S1. Unknown molecular features (identification level of 4 in the ABs and extracellular fluid) with VIP> 2.0.

#	RT (min)	Monoisotopic mass (Da)	Main fragments (MS/MS)	VIP CisPt vs UV	Trend*
**ABs fluid**					
1	0.7	382.1050	203.0546	2.1	↓
2	0.8	158.0192	72.0854, 58.0633, 64.9800, 55.0534	2.2	↓
3	0.9	85.0885	57.0573, 56.0493	2.3	↓
4	0.9	204.1457	86.0944	3.8	↑
5	1.0	145.0730	86.0571, 72.0796, 74.0608	2.9	↓
6	1.0	73.0522	58.0286, 59.0353	6.5	↑
7	1.0	172.0705	86.0962	2.0	↓
8	1.1	159.0682	132.0810, 130.0636	2.5	↓
9	4.7	179.0943	91.0534, 62.0596	2.5	↑
10	6.3	508.2097	465.2330, 175.1161	2.2	↓
11	7.9	514.1619	335.1050, 217.0613, 160.0426	3.0	↓
12	7.9	439.1406	160.0408, 263.1010, 176.0707, 106.0500	2.7	↓
13	8.0	481.1522	160.0360, 187.0704, 148.0612	2.7	↓
14	8.1	514.1618	160.0426, 335.1003, 217.0606	2.7	↓
15	8.1	453.1555	277.1165, 160.0347, 217.0594	2.2	↓
16	8.9	379.1557	176.0713, 176.0713	2.5	↑
17	8.9	449.1612	116.0694, 160.9437, 176.0714	2.5	↓
18	10.2	451.1775	118.0869, 98.0580, 72.0859	2.6	↓
19	10.4	366.1246	160.0439, 217.0635	2.0	↓
20	11.4	465.1926	160.0408, 132.0988, 86.0953	3.6	↓
21	11.5	465.1931	132.1008, 160.0420, 86.0955	3.5	↓
22	12.2	499.1772	160.0431, 166.0846,120.0809, 176.0757	2.6	↓
23	18.6	199.1933	57.0675, 112.9580	2.3	↓
**Extracellular fluid**					
24	0.9	204.1449	86.0951	3.7	↓
25	1.0	145.0732	86.0619	2.7	↑
26	1.0	276.1671	86.0596, 132.1018	2.7	↓
27	1.1	248.1272	123.0802, 81.0417, 221.1392	2.4	↓
28	1.1	159.0681	132.0804, 130.0648, 72.0808	2.4	↓
29	1.6	406.1302	122.0696	2.4	↓
30	2.4	320.1369	285.1276, 138.0504, 146.0648, 117.1022	3.0	↓
31	2.7	283.1051	68.0494, 114.0507, 164.9309	3.5	↓
32	7.1	1202.4052	-	2.2	↓
33	7.8	735.2173	428.0904	2.2	↓
34	8.8	379.1557	160.0430, 176.0697, 217.0611	3.4	↑
35	10.4	424.1296	160.0424, 217.0638	2.0	↑
36	12.4	268.1309	-	2.6	↑
37	15.1	386.1725	105.0729	2.8	↑
38	15.1	526.2786	-	2.7	↑

*↑: The metabolite (on average) is more abundant in CisPt; ↓: The metabolite (on average) is less abundant in CisPt.
